# Comprehensive analysis of PPARγ agonist activities of stereo-, regio-, and enantio-isomers of hydroxyoctadecadienoic acids

**DOI:** 10.1042/BSR20193767

**Published:** 2020-04-23

**Authors:** Aya Umeno, Mami Sakashita, Sakiko Sugino, Kazutoshi Murotomi, Tsugumi Okuzawa, Naoki Morita, Kentaro Tomii, Yuko Tsuchiya, Kazuhiko Yamasaki, Masanori Horie, Kentaro Takahara, Yasukazu Yoshida

**Affiliations:** 1 Health Research Institute, National Institute of Advanced Industrial Science and Technology (AIST), 2217-14 Hayashi-cho, Takamatsu, Kagawa 761-0395, Japan; 2 Bioproduction Research Institute, National Institute of Advanced Industrial Science and Technology (AIST), 2-17-2-1 Tsukisamu-higashi, Toyohira-ku, Sapporo, Hokkaido 062-8517, Japan; 3 Artificial Intelligence Research Center (AIRC), National Institute of Advanced Industrial Science and Technology (AIST), 2-4-7 Aomi, Koto-ku, Tokyo 135-0064, Japan; 4 Biotechnology Research Institute for Drug Discovery, National Institute of Advanced Industrial Science and Technology (AIST), 2-4-7 Aomi, Koto-ku, Tokyo 135-0064, Japan; 5 AIST-Tokyo Tech Real World Big-Data Computation Open Innovation Laboratory (RWBC-OIL), 2-12-1 Ookayama, Meguro-ku, Tokyo 152-8550, Japan; 6 Biomedical Research Institute, National Institute of Advanced Industrial Science and Technology (AIST), 1-1-1 Higashi, Tsukuba, Ibaraki 305-8566, Japan; 7 Thermo Fisher Scientific Japan, 3-9 Moriya-cho, Kanagawa-ku, Yokohama, Kanagawa 221-0022, Japan

**Keywords:** docking simulation, hydroxyoctadecadienoic acid, lipids, luciferase reporter assay, PPARg agonist activity, structural characterization

## Abstract

Hydroxyoctadecadienoic acids (HODEs) are produced by oxidation and reduction of linoleates. There are several regio- and stereo-isomers of HODE, and their concentrations *in vivo* are higher than those of other lipids. Although conformational isomers may have different biological activities, comparative analysis of intracellular function of HODE isomers has not yet been performed. We evaluated the transcriptional activity of peroxisome proliferator-activated receptor γ (PPARγ), a therapeutic target for diabetes, and analyzed PPARγ agonist activity of HODE isomers. The lowest scores for docking poses of 12 types of HODE isomers (9-, 10-, 12-, and 13-HODEs) were almost similar in docking simulation of HODEs into PPARγ ligand-binding domain (LBD). Direct binding of HODE isomers to PPARγ LBD was determined by water-ligand observed via gradient spectroscopy (WaterLOGSY) NMR experiments. In contrast, there were differences in PPARγ agonist activities among 9- and 13-HODE stereo-isomers and 12- and 13-HODE enantio-isomers in a dual-luciferase reporter assay. Interestingly, the activity of 9-HODEs was less than that of other regio-isomers, and 9-(*E,E*)-HODE tended to decrease PPARγ-target gene expression during the maturation of 3T3-L1 cells. In addition, 10- and 12-(*Z,E*)-HODEs, which we previously proposed as biomarkers for early-stage diabetes, exerted PPARγ agonist activity. These results indicate that all HODE isomers have PPARγ-binding affinity; however, they have different PPARγ agonist activity. Our findings may help to understand the biological function of lipid peroxidation products.

## Introduction

Lipid peroxidation has been extensively studied in the fields of chemistry, biology, food science, and medicine [1]. Lipids are oxidized by three distinct mechanisms: (1) enzymatic oxidation; (2) non-enzymatic, free radical-mediated oxidation; (3) non-enzymatic, non-radical-mediated oxidation. Specific oxidation products are yielded via each oxidation mechanism. Polyunsaturated fatty acids (PUFAs) and their esters are readily oxidized by free radical-mediated oxidation, as they have more than two *cis*-double bonds. Linoleates (18:2(n-6)) are the most abundant PUFAs *in vivo.* Their oxidation occurs by a straightforward mechanism, producing oxidation products simpler than those derived from arachidonates (20:4(n-6)), and more highly unsaturated fatty acids, such as docosahexaenoates (22:6(n-3)).

**Figure 1:**
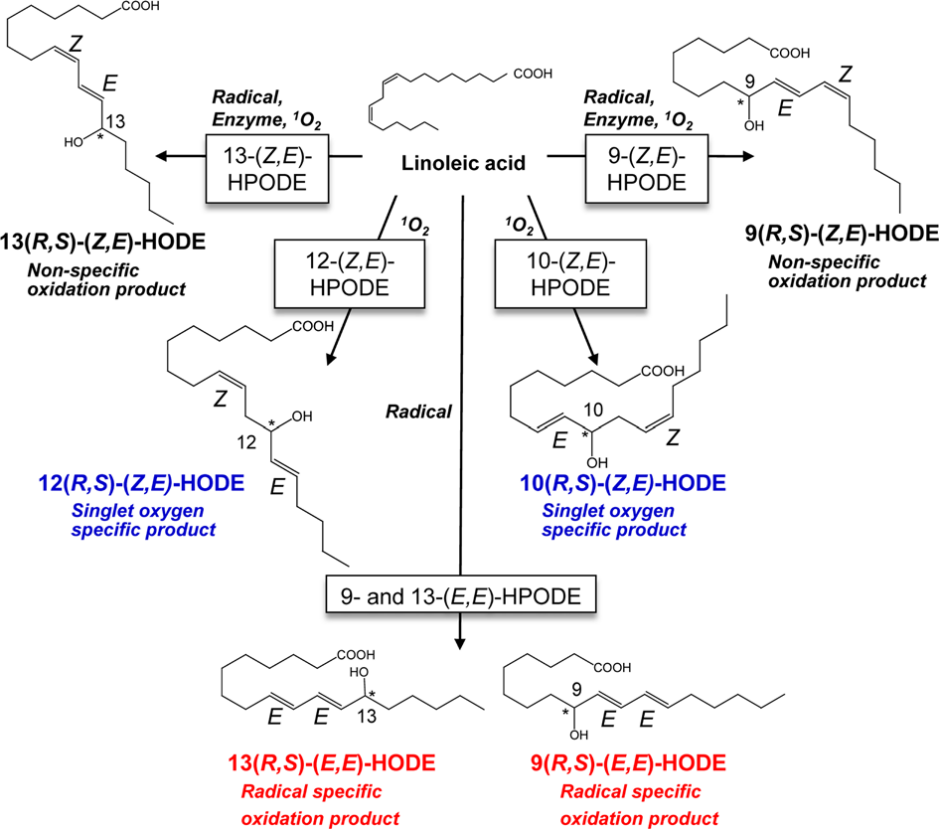
Chemical structure of HODE isomers Different regio-isomers of HODE are produced by different mechanisms, such as radical-mediated oxidation, enzyme-mediated oxidation, and singlet oxygen (^1^O_2_)-mediated oxidation. Blue and red indicate singlet oxygen- and radical-specific products, respectively.

Hydroperoxyoctadecadienoic acids (HPODEs) are obtained from abundant parent lipids, such as linoleates, by simple mechanisms. They are reduced readily to form hydroxyoctadecadienoic acids (HODEs). The HPODEs that are formed by free radical-mediated oxidation comprise four racemic isomers: 13-hydroperoxy-9(*Z*), 11(*E*)-octadecadienoic acid (13-(*Z,E*)-HPODE); 13-hydroperoxy-9(*E*), 11(*E*)-octadecadienoic acid (13-(*E,E*)-HPODE); 9-hydroperoxy-10(*E*), 12(*Z*)-octadecadienoic acid (9-(*E,Z*)-HPODE); 9-hydroperoxy-10(*E*), 12(*E*)-octadecadienoic acid (9-(*E,E*)-HPODE) (Figure 1). Of these, 9- and 13-(*Z,E*)-HPODEs are also formed by enzymatic oxidation via lipoxygenase as enantio-, regio-, and stereo-specific products, thus making 9- and 13-(*E,E*)-HPODE radical-specific products. In contrast, singlet oxygen oxidizes linoleic acids by non-radical-mediated oxidation to form 13-(*Z,E*)-HPODE, 10-hydroperoxy-8(*E*), 12(*Z*)-octadecadienoic acid (10-(*E,Z*)-HPODE), 12-hydroperoxy-9(*Z*), 13(*E*)-octadecadienoic acid (12-(*Z,E*)-HPODE), and 9-(*E,Z*)-HPODE. 10- and 12-(*Z,E*)-HPODEs are singlet oxygen-specific products. As HODEs, the reduced forms of HPODEs, are stable under physiological conditions, we developed a method to measure HODE isomers in the same sample, including 9-(*Z,E*) and 9-(*E,E*)-HODE, 10-(*Z,E*)-HODE, 12-(*Z,E*)-HODE, and 13-(*Z,E*) and 13-(*E,E*)-HODE, to understand the oxidation mechanism *in vivo* [2,3]. Our measurement revealed that HODE isomers can be prominent biomarkers for some diseases [4–7]. For example, plasma levels of 10- and 12-(*Z,E*)-HODEs, which are singlet oxygen-specific products, are significantly correlated with glucose levels in patients with early-stage type 2 diabetes [6,7].

Several lipid peroxidation products serve as signaling factors and consequently contribute to cytotoxicity or cytoprotection [1,8,9]. 4-Hydroxynonenal, which is a major product derived from *n*−*6* PUFA oxidation, can affect cell fate, either to cell death or survival, depending on metabolic state and cell type [10]. The structural features of some lipids affect their biological activity *in vivo*. For example, palmitoleic acid isomers (16:1*n*−*7*, 16:1*n*−*9*, 16:1*n*−*10*) manifest anti-inflammatory activity at different concentrations in phagocytic cells [11]. Our findings also led to the speculation that physiological activities are divergent among HODE isomers, because the levels of only 10- and 12-(*Z,E*)-HODEs are correlated with plasma glucose level in the prediabetic state [6,7]. However, such variation among the stereo-, regio-, and enantio-isomers of HODE is not yet clarified.

Peroxisome proliferator-activated receptor γ (PPARγ) belongs to the nuclear receptor superfamily and serves as a receptor for various metabolites of fatty acids, including 5-hydroxyeicosatetraenoic acid, 15-deoxy-Δ12,14-prostaglandin J2, and 13-oxo-octadecadienoic acid [12]. PPARγ transcriptionally regulates adipocyte differentiation, glucose homeostasis, insulin sensitivity, and inflammation [13]. The intracellular signaling pathways mediated by PPARγ have been considered therapeutic targets for type 2 diabetes and Alzheimer’s disease [13,14]. In fact, the PPARγ agonist pioglitazone (PGZ), a thiazolidinedione (TZD) derivate, is used for the treatment of type 2 diabetes [15]. HODEs are abundant lipid metabolites in the plasma, as we can measure HODE isomers in preadipocyte 3T3-L1 cells (data not shown) and human and mouse plasma using LC-MS/MS [4,6]. Thus, HODE isomers may be endogenous ligands for PPARγ. However, the effects of HODE isomers on PPARγ ligand binding activity are not completely understood.

In the present study, we comprehensively investigated whether there are differences in PPARγ agonist activity among HODE isomers in terms of structural features and transcriptional activity. In ligand–receptor docking simulation, PPARγ could accommodate twelve types of HODE isomers with almost similar binding affinities. We also revealed that not only 9- and 13-HODEs, but also 10- and 12-HODEs could directly bind to PPARγ in water-ligand observed via gradient spectroscopy (WaterLOGSY) NMR. PPARγ activities determined by dual-luciferase reporter assay were different among stereo-, regio-, and enantio-isomers of HODEs. Interestingly, 10- and 12-(*Z,E*) and 13-(*E,E*) forms of HODEs induce PPARγ-mediated transcriptional activation, whereas 9-(*E,E*)-HODE down-regulates PPARγ-target gene expression in preadipocyte 3T3-L1 cells. Our results demonstrate for the first time that 9-, 10-, 12-, and 13-HODE isomers have PPARγ-binding activity, but that the PPARγ agonist activity is different among HODE stereo-, regio-, and enantio-isomers.

## Materials and methods

### Materials

Lipid peroxidation products were procured from Cayman Chemical Company (Ann Arbor, MI, U.S.A.). The racemic isomers 13-(*Z,E*)-HODE, 9-(*Z,E*)-HODE, 13-(*E,E*)-HODE, 9-(*E,E*)-HODE, 10-(*Z,E*)-HODE, 12-(*Z,E*)-HODE, and 13(*R*)- and 13(*S*)-(*Z,E*)-HODEs were procured from Larodan Fine Chemicals (Malmo, Sweden). The other materials used were of the highest grade commercially available. The fractionation of 10(*R*)- and 10(*S*)-(*Z,E*)-HODEs and 12(*R*)- and 12(*S*)-(*Z,E*)-HODEs was performed by Reprosil Chiral NR (250 mm × 2 mm, 8 mm; Dr. Maisch, Ammerbuch, Germany); the compounds were eluted at 0.3 ml/min with 97% hexane/3% isopropanol/0.01% acetic acid [16], and their chiral structure was confirmed.

### Preparation of initial complex structures for docking simulations

For reasons mentioned in the *Results* section, two crystal structures [Protein Data Bank (PDB) IDs: 2PRG [17] and 5YCP [18]] of the PPARγ ligand-binding domain (LBD) in complex with the co-activator SRC1 and rosiglitazone, a member of the TZD class of antidiabetic drugs, were used to construct initial complex structures for docking simulations. We also referred to the structure of the PPARγ LBD in complex with 9(*S*)-HODEs [PDB ID: 2VSR [19]] to gain insight into the binding geometries of C18 fatty acids.

Superimposing the structures of PPARγ LBD in 2PRG and 5YCP on the structure of the chain A in 2VSR showed that the oxygen atoms in the two carbonyl groups of the TZD group of rosiglitazone in both 2PRG and 5YCP almost overlapped with those in the carboxylate group of (the first) one of the two 9(*S*)-HODEs bound to the chain A in 2VSR. In addition, the researchers who determined these structures have mentioned that S289, H323, H449, and Y473 of PPARγ form hydrogen bonds with the carbonyl (in 2PRG and 5YCP) and carboxylate (in 2VSR) groups. We constructed two initial complex structures for docking simulations, based on the PPARγ LBD with SRC1 from 2PRG and 5YCP. These initial structures were constructed by superimposing the PPARγ LBD in 2PRG and 5YCP on that in 2VSR, and by replacing rosiglitazone with the first 9(*S*)-HODE of the chain A in 2VSR. All 12 enantiomers of HODEs, 9(*R*)-(10*E*,12*E*), 9(*S*)-(10*E*,12*E*), 9(*R*)-(10*E*,12*Z*), 9(*S*)-(10*E*,12*Z*), 10(*R*)-(8*E*,12*Z*), 10(*S*)-(8*E*,12*Z*), 12(*R*)-(9*Z*,13*E*), 12(*S*)-(9*Z*,13*E*), 13(*R*)-(9*E*,11*E*), 13(*S*)-(9*E*,11*E*), 13(*R*)-(9*Z*,11*E*), and 13(*S*)-(9*Z*,11*E*), were constructed manually by using the Builder tool in Molecular Operating Environment (MOE) [20]. All the models were then energetically minimized to obtain stable conformations.

### Flexible docking of HODEs into PPARγ

We performed ‘template docking’ using MOE for each of the 12 HODE models mentioned above (target HODEs) and their initial complex structures with 2PRG and 5YCP. The initial complex structure, including the PPARγ LBD with SRC1 and 9(*S*)-HODE (as a template HODE), and each of the 12 HODE models were respectively loaded into MOE. Then, the loaded structures were refined and hydrogen atoms were added to them by the QuickPrep tool. To perform template docking, we selected one carbon and two oxygen atoms from the carboxylate group of the template HODE in the Query item. This procedure led to the replacement of the template HODE (originated from 2VSR) with a target HODE (constructed by MOE) at the beginning of the docking. It should be noted that the positions of these carbon and oxygen atoms were fixed during docking. We performed ‘Induced Fit’, which allows side chains of the ligand-binding pocket and the target HODE to be flexible, excluding the carbon and oxygen atoms mentioned above. However, other side chains and main chains remain fixed. We set ‘GBVI/WSA dG’ for energy calculations, and stored docking poses up to 30 best scores for both the placement and refinement steps. The obtained docking poses were ranked based on the final score S, which was estimated from the scores calculated in all stages during docking simulation.

Current methods are usually based on docking a single ligand at a time. Thus, when there are two ligands at a time, they have to be re-docked using the ligand-bound protein. This makes it more difficult to obtain ‘correct’ scores and poses than the cases of single ligands. Therefore, in the present study, we focused only on a single molecule of HODE.

### NMR analyses

For evaluation of direct binding of HODEs to PPARγ, 0.1 mM of each HODE isomer (9-(*Z,E*)-HODE, 9-(*E,E*)-HODE, 10-(*Z,E*)-HODE, 12-(*Z,E*)-HODE, 13-(*Z,E*)-HODE, 13-(*E,E*)-HODE) and 2.0 μM PPARγ LBD (BioVision, CA, U. S. A.) were dissolved in 20 mM potassium phosphate (pH 7.4), 50 mM KCl, 1.0 mM d_10_-dithiothreitol (Isotech, Inc.), 0.1 mM sodium 2,2-dimethyl-2-silapentane-5-sulfonate (DSS; Sigma–Aldrich), and 5% D_2_O. Measurements of WaterLOGSY [21] were carried out using an Avance III 500 spectrometer (Bruker BioSpin; 500.13 MHz) at 35°C, with a mixing time of 2 s. We also collected WaterLOGSY spectra of samples without PPARγ LBD, which were subtracted from those of samples containing the protein in the difference spectra. Chemical shifts were referenced to the internal DSS. Signal assignments of the methyl, methylene, and methine protons were temporarily made according to a similar compound, linoleic acid, registered in the Biological Magnetic Resonance Data Bank (http://www.bmrb.wisc.edu) (Supplementary Figure S1).

### PPARγ agonist activity

PPARγ agonist activity was evaluated by the reporter gene assay using a method slightly modified from that reported previously [22]. Briefly, CV-1 cells derived from African green monkey kidney (Japanese Collection of Research Bioresources (JCRB) Cell Bank, Osaka, Japan), which are highly susceptible to SV40 infection and have been used in luciferase reporter assays, were seeded at a concentration of 2 × 10^5^ per well in 6-well plates and cultured overnight in Dulbecco’s Modified Eagle’s Medium (DMEM) supplemented with 10% charcoal-treated and heat-inactivated fetal bovine serum (FBS). The cells in each well were transiently transfected with 1 μg of the expression plasmid pGal4DBD/PPARγLBD and 0.9 μg of pGal4-Luc, which encodes the luciferase reporter gene Fluc (firefly luciferase), together with 0.1 μg of pGL4.75hRluc-CMV, which encodes the luciferase reporter gene hRluc (*Renilla reniformis* luciferase). Transfection was performed using X-tremeGENE HP DNA Transfection Reagent (Sigma–Aldrich, St. Louis, MO, U.S.A.). After 6 h of incubation, the cells were harvested and treated with trypsin, and then 1.6 × 10^4^ cells were dispensed per well in a 96-well plate with fresh DMEM containing each concentration of HODEs. We used 10 μM PGZ as a positive control and 0.5% dimethyl sulfoxide as a negative control. After 48 h of incubation, the cells were washed with phosphate-buffered saline, and the expression of the reporter gene was indicated by measuring the activity of Fluc and hRluc using a luminometer (Luminescencer, AB-2350EX, ATTO, Tokyo, Japan). PPARγ agonist activity was determined by the activity of Fluc corrected for transfection efficiency based on the activity of the internal control hRluc; this system is known as ‘dual-luciferase reporter assay system’ (Promega, Madison, WI, U.S.A.). Results are expressed as sigmoidal dose–response curves fitting using Origin software (LightStone, Tokyo, Japan).

### Cell culture

Mouse 3T3-L1 preadipocytes were purchased from the American Type Culture Collection (ATCC, Manassas, VA, U.S.A.) and cultured in DMEM supplemented with 10% FBS at 37°C in a humidified atmosphere of 5% CO_2_. To differentiate 3T3-L1 into mature adipocytes, the cells were seeded into 6-well plates (Nunc, Roskilde, Denmark) at a concentration of 8 × 10^4^ cells per well, and the medium was replaced after 2 days. At 2 days after confluence, 3T3-L1 cells were transferred to adipogenic differentiation medium, which is DMEM containing 10% FBS and AdipoInducer Reagent (10 μg/ml insulin, 2.5 μM dexamethasone, and 0.5 mM 3-isobutyl-1-methylxanthine; Takara Bio Inc., Shiga, Japan), for 2 days. After that, the cells were cultured in adipocyte maintenance medium, which is DMEM containing 10% FBS and 10 μg/ml insulin, for 2 days. To evaluate the effect of HODE isomers in 3T3-L1 cells, the cells were cultured in the medium containing 12 μg/ml HODEs throughout the experiment.

### Isolation of total RNA and quantitative real-time PCR

3T3-L1 cells were harvested using TRIzol reagent (Invitrogen, CA, U.S.A.), and the total RNA was isolated and purified using the RNeasy Mini kit (Qiagen, Hilden, Germany), according to the manufacturer’s instructions. The total RNA (500 ng) was used as a template for cDNA synthesis using ReverTra Ace qPCR RT Master Mix with gDNA Remover (TOYOBO, Osaka, Japan). Gene expression was analyzed with the KOD SYBR qPCR Mix (TOYOBO) using a CFX Connect Real-Time System (Bio-Rad, Hercules, CA, U.S.A.). The following primers were used for qPCR: 5′-CAGCCTTTCTCACCTGGAAG-3′ and 5′-TTGTGGCAAAGCCCACTC-3′ for mouse aP2; 5′-GAGTCGGCCGACTTCTACG-3′ and 5′-GTCTCGTGCTCGCAGATGC-3′ for mouse C/EBPα; 5′-GGGAGTTTGGCTCCAGAGTTT-3′ and 5′-TGTGTCTTCAGGGGTCCTTAG-3′ for mouse Lpl; 5′-TGCCGAAGATGACGTTACTACAA-3′ and 5′-CCATCCAACCTGCACAAGTTC-3′ for mouse Adipoq; 5′-AGATGGAGGAGTTCGTGTATAAG-3′ and 5′-ATGTAGCAGGTAGTCGTTGTC-3′ for mouse AdipoR1; 5′-TTCTTTGCAGCTCCTTCGTT-3′ and 5′-GACCAGCGCAGCGATATC-3′ for mouse β-actin. The PCR conditions were as follows: initial denaturation at 98°C for 2 min, followed by 40 cycles of 98°C for 10 s, 60°C for 10 s, and 68°C for 30 s. The melting curve was then analyzed at a linear temperature gradient from 65 to 95°C to assess whether a single PCR product was synthesized. Relative gene expression levels were determined after normalization to the expression level of β-actin in corresponding samples.

### Statistical analysis

The results are expressed as mean ± standard deviation (SD). Statistical analysis was performed using analysis of variance (ANOVA) followed by Tukey’s test for multiple comparisons using the Ekuseru–Toukei 2012 software (Social Survey Research Information Co., Ltd., Tokyo, Japan). Results with *P*<0.05 were considered significant.

## Results

### Docking simulation of HODE isomers in PPARγ ligand-binding pocket

At first, to examine whether PPARγ can accommodate stereo-, regio-, and enantio-isomers of HODE in the ligand-binding pocket, we performed docking simulations of 9-, 10-, 12-, and 13-HODEs with PPARγ. We prepared two initial complex structures based on 2PRG and 5YCP for confirmation. As described in *Materials and methods* section, we used the structures of PPARγ LBD in complex with the co-activator SRC1 in 2PRG and 5YCP. In both PDB entries (2PRG and 5YCP), PPARγ originally binds to rosiglitazone. 9(*S*)-HODE, which is derived from 2VSR, was used as a template HODE. Each of the enantiomer models of 9-, 10-, 12-, and 13-HODE was constructed manually, and was put in the ligand-binding pocket of PPARγ in the initial complex structure on MOE, so that the carboxylate group in a target HODE overlapped with that of the template 9(*S*)-HODE.

**Figure 2:**
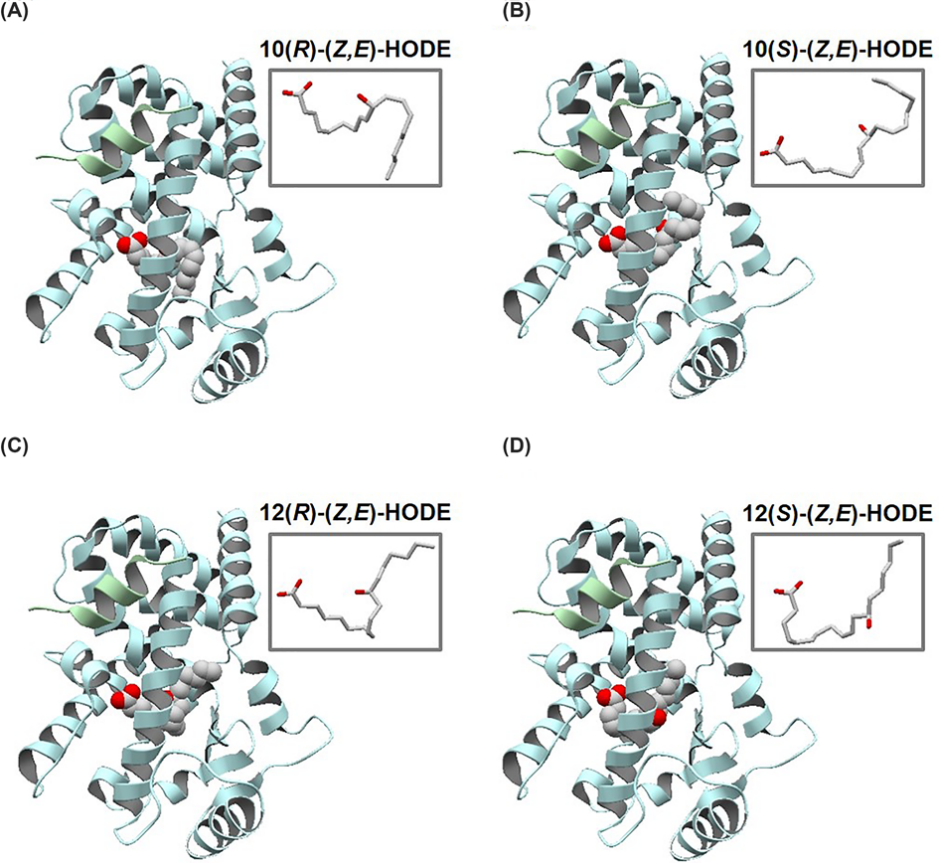
Overall structures of PPARγ complex with HODE isomers Docking poses of (**A**) 10(*R*)-(*Z,E*)-, (**B**) 10(*S*)-(*Z,E*)-, (**C**) 12(*R*)-(*Z,E*)-, and (**D**) 12(*S*)-(*Z,E*)-HODEs with the best *S* score (shown in Table 1), based on PPARγ protein from 2PGR. PPARγ and SRC1 are colored light blue and green, respectively, and HODEs are shown in space-fill model. The conformations of HODEs with the best S scores are shown in stick models at upper-right regions.

The best scores for docking poses of target HODEs for the initial complex structures based on 2PRG and 5YCP are shown in Table 1. We found that the best scores for target HODEs were comparable with each other. We recognized the locations of the oxygen atoms of hydroxy groups around the middle of 10- and 12(*R, S*)-HODEs (Figure 2) wherein the best scores were similar to those of hydroxy groups in the template HODE and of ethoxy group of rosiglitazone in 2PRG and 5YCP. These results suggest that PPARγ can accommodate HODEs with similar binding affinities regardless of the stereo- and enantio-isomers. Further, the conformations around the hydroxy groups of 9-, 10-, 12-, and 13-HODEs can be stabilized in the ligand-binding pocket in a manner similar to that in 2PRG, 5YCP, and 2VSR. According to these observations, we concluded that 10- and 12-HODEs have an ability to bind to PPARγ.

**Table 1:** The best *S* scores of docking poses based on PPARγ of 2PRG and 5YCP

Compound	Best *S* score	
	2PRG	5YCP
9(*R*)-(*E,E*)-HODE	−8.62	−9.18
9(*S*)-(*E,E*)-HODE	−8.88	−9.13
9(*R*)-(*Z,E*)-HODE	−8.56	−8.86
9(*S*)-(*Z,E*)-HODE	−8.84	−8.95
10(*R*)-(*Z,E*)-HODE	−8.67	−8.84
10(*S*)-(*Z,E*)-HODE	−8.82	−9.04
12(*R*)-(*Z,E*)-HODE	−8.33	−9.11
12(*S*)-(*Z,E*)-HODE	−8.76	−9.33
13(*R*)-(*E,E*)-HODE	−8.24	−8.99
13(*S*)-(*E,E*)-HODE	−8.46	−8.89
13(*R*)-(*Z,E*)-HODE	−8.83	−9.08
13(*S*)-(*Z,E*)-HODE	−8.73	−8.93

### Interaction of HODE isomers with PPARγ ligand-binding domain

**Figure 3:**
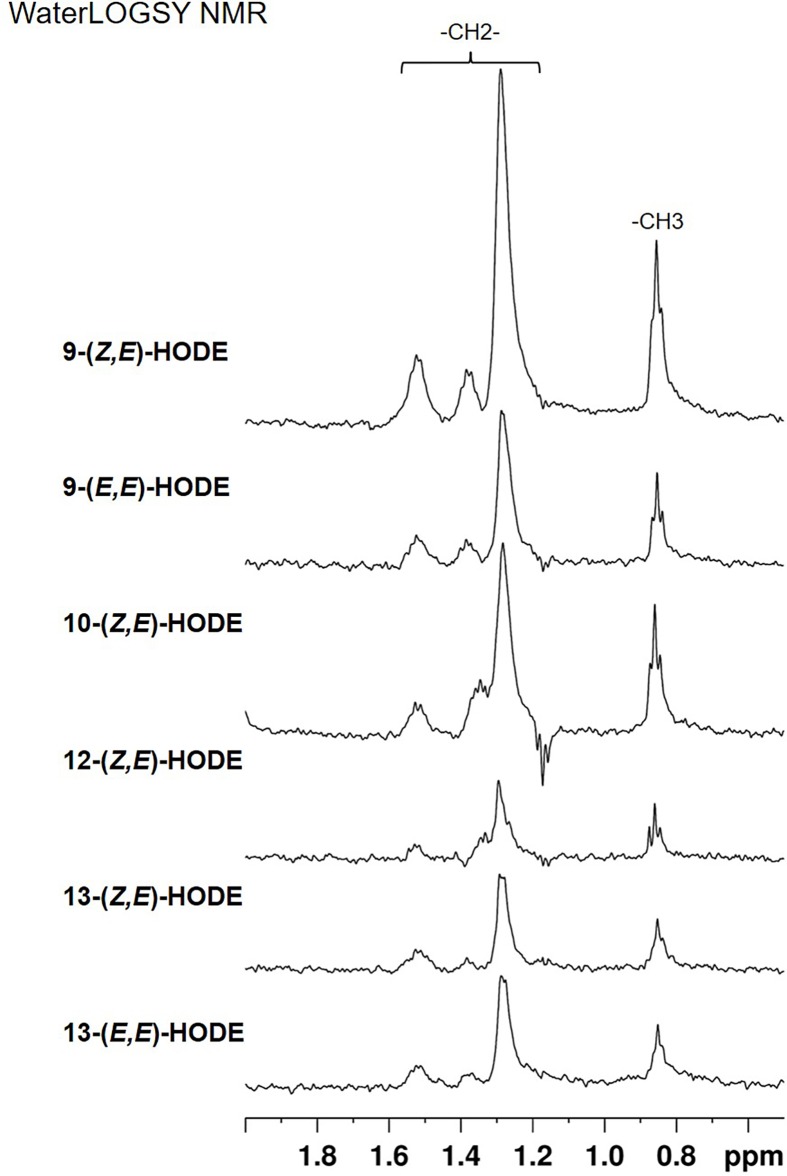
WaterLOGSY spectra of HODE isomers in the methyl/methylene region Shown are the difference spectra wherein those in the absence of PPARγ LBD were subtracted from those in the presence of the protein (see Supplementary Figure S2). From top to bottom, spectra for 9-(*Z,E*)-HODE, 9-(*E,E*)-HODE, 10-(*Z,E*)-HODE, 12-(Z,E)-HODE, 13-(*Z,E*)-HODE, and 13-(*E,E*)-HODE are presented.

To clarify whether HODEs can directly bind to PPARγ, we employed WaterLOGSY NMR, which is a popular method for identification of small compounds interacting with macromolecules [21]. In the WaterLOGSY experiment, small compounds that interact with the target protein exhibit peaks with signs opposite (positive or negative) that of free compounds. This was indeed the case with all the HODE isomers, for which the WaterLOGSY spectra were recorded in the presence and absence of PPARγ LBD (Supplementary Figure S2). The effects of binding were evaluated in the difference spectra, showing clear peaks for all the isomers (Figure 3). The results confirmed that all the HODE isomers directly interact with PPARγ LBD. It should be noted, however, that the peak intensities were different among the isomers. This suggested that the binding affinity and/or the geometry of binding, including those of bound water, are affected by the stereochemistry of HODEs.

### Analysis of PPARγ agonist activities of HODE isomers

**Figure 4:**
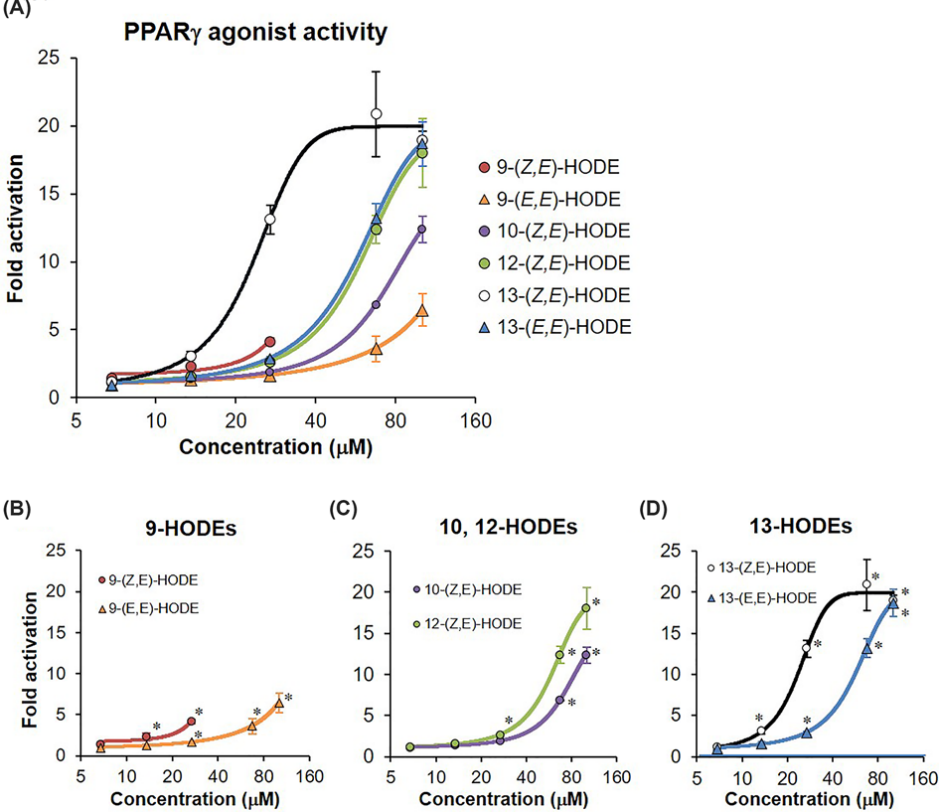
PPARγ agonist activity of racemic HODE isomers (**A**) All HODE isomers, (**B**) 9-HODEs, (**C**) 10- and 12-HODEs, and (**D**) 13-HODEs. The agonist activity of PPARγ was evaluated using the dual-reporter assay system. PPARγ agonist PGZ and 0.05% dimethyl sulfoxide (DMSO) were used as a positive control and negative control, respectively to confirm whether the luciferase assay was successful. Amounts of added HODEs were 6.8, 13.5, 27.0, 67.5, and 101.2 μM, respectively. All values are presented as mean ± SD of three replicates (*n*=3 each). The activity is indicated as fold change with respect to the degree of PPARγ agonist activity in negative control, which is set to 1. *P<0.05 compared with the negative control. Results for >27 μM 9-(*Z,E*)-HODE that induced cellular damage, which indicated marked reduction of luminescence intensity in the internal control, were not plotted.

We evaluated PPARγ agonist activities of HODE isomers using the luciferase reporter assay. The concentration-dependent activities of racemic isomers of HODE are shown in Figure 4. As per a preliminary test, >135 μM (40 μg/ml) HODEs exerted cytotoxicity; thus, we measured their activities at concentrations <101.2 μM (30 μg/ml). The ratio of luciferase activities was increased in dose-dependent manner when CV-1 cells were treated with racemic isomers of HODE (Figure 4A), except for 9-(*Z,E*)-HODE, which had cytotoxicity at 67.5 and 101.2 μM (Figure 4A,B), in CV-1 cells. The relative luminescence intensities in treatments with 10-(*Z,E*)-, 12-(*Z,E*)-, 13-(*Z,E*)-, and 13-(*E,E*)-HODEs at a concentration of 101.2 μM were ten-fold higher than those in the negative control (Figure 4A,C,D). In contrast, the intensity in treatment with 9-(*E,E*)-HODE at the same concentration was 6.5-fold higher (Figure 4A,B). These dose-dependent effects of HODE isomers on relative luminescence intensities were consistently observed in two independent experiments. The *Z,E* form of 13-HODE, which is yielded by the enzymatic, free radical-mediated, and singlet oxygen-mediated oxidation, tended to exert higher PPARγ activity than the corresponding *E,E* form, which is yielded by free radical-mediated oxidation (Figure 4D).

**Figure 5:**
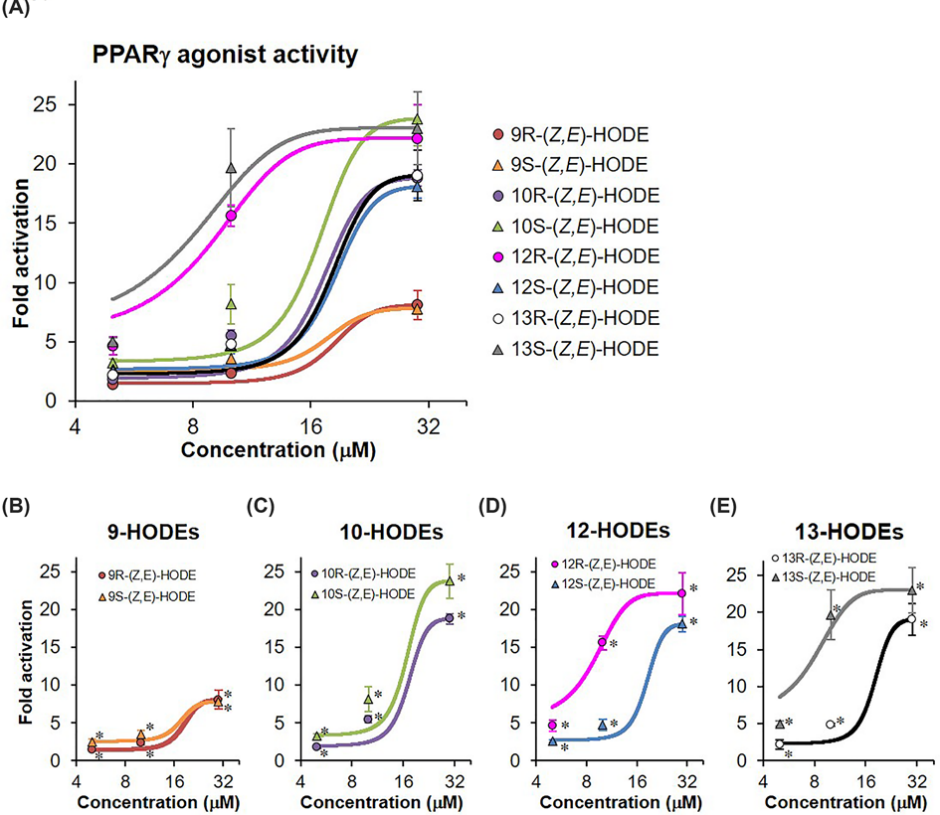
PPARγ agonist activity of HODE enantio-isomers (**A**) all HODE isomers and (**B**) 9-HODEs, (**C**) 10-HODEs, (**D**) 12-HODEs, and (**E**) 13-HODEs. The agonist activity of PPARγ was evaluated by the dual-reporter assay system. PPARγ agonist PGZ and 0.05% dimethyl sulfoxide (DMSO) were used as positive and negative controls, respectively. Amounts of added HODEs were 5, 10, and 30 μM, respectively. All values are presented as mean ± SD of three replicates (*n*=3 each). The activity is indicated as fold change with respect to the degree of PPARγ agonist activity in negative control, which is set to 1. *P<0.05 compared with the negative control.

Furthermore, we investigated whether enantiomeric differences in HODEs affect PPARγ ligand-binding activity. As shown in Figure 5A, luciferase activities were significantly increased in all (*Z,E*)-HODEs at concentrations of 5–30 μM. The activities of both 9(*R*) and 9(*S*)-(*Z,E*)-HODEs tended to be lower than those of other HODE isomers (Figure 5A), which was consistent with the observations of racemic isomers (Figure 4). 9(*R*) and 9(*S*)-(*Z,E*)-HODEs had no cytotoxic effect when examined individually, though the racemic mixture of 9-(*Z,E*)-HODE induced cytotoxicity at 67.5 and 101.2 μM (Figure 4B). There were marked differences in activities between the (*R*) and (*S*) forms of 12- and 13-HODE isomers (Figure 5D,E), whereas the activities between the (*R*) and (*S*) forms of 9- and 10-HODE were almost the same (Figure 5B,C). These results indicate that PPARγ agonist activities are different in stereo- and enantio-isomers of HODEs, and that the activity of 9-HODEs was lower than that of other regio-isomers, including 10-, 12-, and 13-HODEs.

### Effect of HODE isomers on the expression level of PPARγ-target gene in 3T3-L1 cells

**Figure 6:**
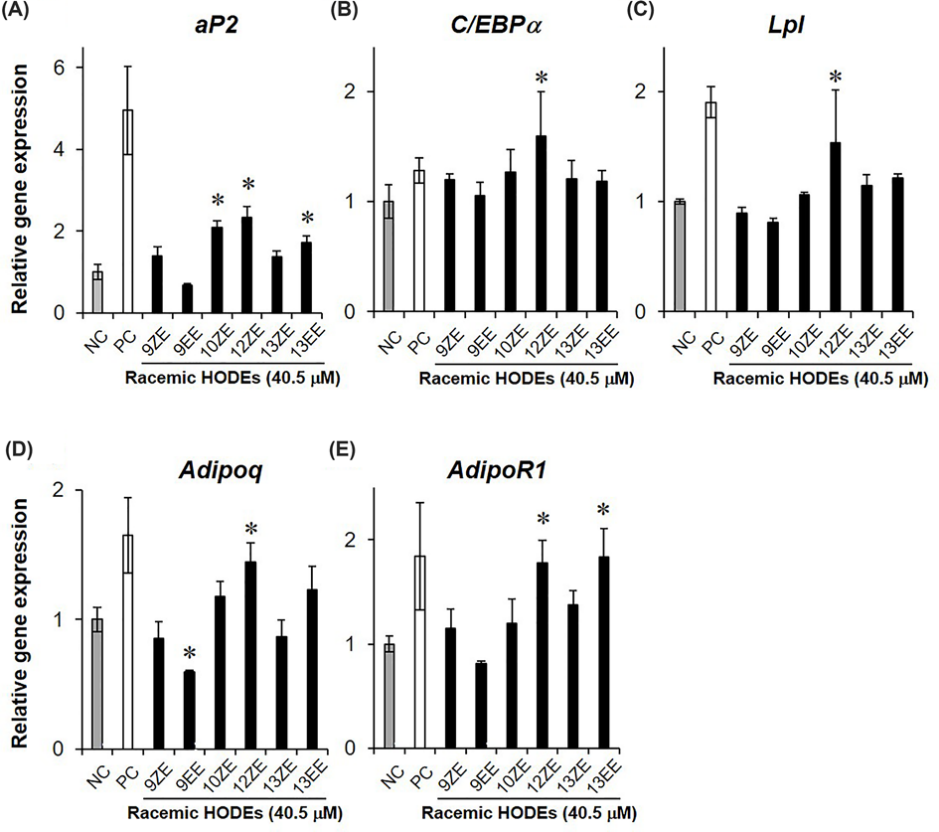
PPARγ-target gene expression in differentiated 3T3-L1 cells with or without HODE isomers The expression levels of (**A**) *aP2*, (**B**) *C/EBPα*, (**C**) *Lpl*, (**D**) *Adipoq*, and (**E**) *AdipoR1* were measured at 4 days after the start of 3T3-L1 differentiation. All racemic mixtures of HODEs (equal amounts of (*R*) and (*S*) forms), are indicated as black bars; 10 μM PGZ and 0.1% DMSO were used as positive control (PC, white bar) and negative control (NC, gray bar), respectively. Results are expressed as mean values relative to control ± SD (*n*=4 each). *P<0.05 compared with NC. 9ZE, 9-(*Z,E*)-HODE; 9EE, 9-(*E,E*)-HODE; 10ZE, 10-(*Z,E*)-HODE; 12ZE, 12-(*Z,E*)-HODE; 13ZE, 13-(*Z,E*)-HODE; 13EE, 13-(*E,E*)-HODE.

PPARγ agonist up-regulates several genes highly expressed in mature adipocytes, including *aP2* (*Fabp4*), *C/EBPα*, and *Lpl* [23]. In addition, adiponectin (*Adipoq*) and its receptor (*AdipoR1*) are up-regulated during the process of preadipocyte 3T3-L1 maturation [24,25]. To investigate whether HODE isomers induce PPARγ-target gene expression, we measured the expression levels of *aP2, C/EBPα, Lpl, Adipoq*, and *AdipoR1* in 3T3-L1 cells, which are often used as an evaluation tool for PPARγ agonist activity in natural products [26], with or without HODEs treatment. At 4 days after induction of differentiation into mature adipocytes, the expression levels of all genes were up-regulated by treatment with the PPARγ agonist PGZ (Figure 6). The expression level of *aP*2, which is a preadipocyte differentiation marker, was significantly increased by treatment with 10- and 12-(*Z,E*)-HODE and 13-(*E,E*)-HODE (Figure 6A). *C/EBPα, Lpl*, and *Adipoq* levels were significantly up-regulated only by the treatment with 12-(*Z,E*)-HODE (Figure 6B–D), and the levels of *AdipoR1* were up-regulated by 12-(*Z,E*)-HODE and 13-(*E,E*)-HODE treatment (Figure 6E). On the contrary, only 9-(*E,E*)-HODE tended to decrease PPARγ-target gene expression compared with the control. Further, *adipq* expression levels were significantly reduced by 9-(*E,E*)-HODE treatment (Figure 6D), although 9-(*E,E*)-HODE had no effect on the expression levels of β-actin, which is an internal control in 3T3-L1 cells (data not shown). These results indicate that the 10- and 12-(*Z,E*) and 13-(*E,E*) forms of HODE induce PPARγ-mediated transcriptional activation during lipid accumulation in 3T3-L1 cells, whereas 9-(*E,E*)-HODE has the opposite effect compared with other HODE isomers.

## Discussion

In the present study, we demonstrated that different HODE isomers have distinct PPARγ transcriptional activities; 10-, 12-, and 13-HODEs significantly increase PPARγ-mediated transcriptional activity, whereas 9-HODEs reduce the transcriptional activity. Furthermore, there were differences in PPARγ agonist activity between stereo-isomers of 9- and 13-HODEs and enantio-isomers of 12- and 13-HODEs. All isomers were able to directly bind to PPARγ LBD. Notably, this is the first study showing that 10- and 12-(*Z,E*)-HODEs, which are specifically produced by singlet oxygen, also have biological activity as PPARγ agonists.

Linoleates are more stable than other PUFAs, including arachidonates and docosahexaenoates, in terms of free radical-mediated oxidation. We have previously shown that HODEs, which are linoleate oxidation products, can be used as biomarkers for the assessment of oxidative stress *in vivo* [2]. There are differences in pharmacological activities between two enantiomers of a chiral drug, such as propranolol and verapamil [27]. We therefore investigated the effects of (*R*) and (*S*) forms of HODE isomers obtained by chiral column chromatography on PPARγ activity. As expected, PPARγ ligand-binding activities between (*R*) and (*S*) forms of 12- and 13-HODE isomers were clearly distinct (Figure 5). On the other hand, there were no marked differences in PPARγ ligand-binding activity between (*R*) and (*S*) forms of 9- and 10-(*Z,E*)-HODEs. The majority of racemic drugs have a major active enantiomer (eutomer) and an inactive or less active enantiomer (distomer) [27]. Other categorized racemic drugs have an equivalent pharmacological activity between the two enantiomers [27]. It is noteworthy that the PPARγ agonist activity of the (*R*) form was higher than that of the (*S*) form in 12-(*Z,E*)-HODE, and conversely, the activity of the (*S*) form was higher than that of the (*R*) form in 13-(*Z,E*)-HODE (Figure 5). In addition, we found that the *Z,E*-forms of 9- and 13-HODEs displayed higher PPARγ ligand-binding activities than the *E,E*-forms (Figure 4). The reason for the difference in PPARγ ligand-binding activities between the (*R*) and (*S*) forms according to regio-isomers and *Z,E-* and *E,E-*forms is unclear. Thus, a greater understanding of the biological function of each HODE isomer requires further studies. PPARγ can accommodate two molecules of 9- and 13-HODEs in the ligand-binding pocket, and binds covalently to conjugated linoleic acids, such as 9- and 13-HODEs [19]. However, there was no evidence of the binding state between non-conjugated fatty acids 10- and 12-HODEs and PPARγ. Therefore, further studies, such as crystal structure analysis, are needed to reveal the extent of direct binding affinity and geometry of binding between HODE isomers and PPARγ LBD. At least, our findings suggest that the mechanism underlying the functional differences in the proliferation rates of colorectal cancer cells between the (*R*) and (*S*) forms of 13-(*Z,E*)-HODE [28] may be due to the differential response of enantio-isomers of 13-(*Z,E*)-HODE to PPARγ-mediated signaling, as the proliferation and apoptosis of cancer cells are regulated by PPARγ [29].

We previously found that only 10- and 12-(*Z,E*)-HODEs out of 12 lipid peroxidation products were significantly correlated with plasma glucose levels in prediabetic patients [6,7]. Moreover, increased plasma levels of 10- and 12-(*Z,E*)-HODEs were observed in obese mice with prediabetic-stage type 2 diabetes [4]. However, there is no report on the role of 10- and 12-(*Z,E*)-HODEs in the development of obesity and diabetes. Herein, we showed that 10- and 12-(*Z,E*)-HODEs induce PPARγ activation, which contributes to improving blood glucose levels by suppressing insulin resistance and inflammation [30], even in preadipocyte 3T3-L1 cells. In addition, 10- and 12-(*Z,E*)-HODEs induce expression of antioxidant genes, such as hemeoxygenase-1 and glutathione peroxidase-2, which is mediated by nuclear translocation of nuclear factor-erythroid 2 p45-related factor 2 (Nrf2) [31]. It is well known reactive oxygen species induce oxidative damage before the pathogenesis of diabetes [32,33]. Therefore, increased plasma levels of 10- and 12-HODEs in the prediabetic state may be a biological response to maintain redox homeostasis in the body to prevent the progression of diabetes.

Interestingly, only 9-HODE showed less PPARγ agonist activity in the luciferase reporter assay than the other regio-isomers (Figures 4A and 5A), although the 9-hydroxyl group displayed a binding affinity for PPARγ LBD similar to other hydroxyl groups (Figure 3 and Table 1). The high peak intensity of 9-(*Z,E*)-HODE in WaterLOGSY NMR suggests that the binding affinity and/or geometry of binding between the isomer and PPARγ LBD may be different from those of other HODE isomers (Figure 3). In addition, treatment with 9-(*E,E*)-HODE can decrease the expression levels of PPARγ-target genes in 3T3-L1 cells (Figure 6). These findings indicate that 9-(*E,E*)-HODE is possibly a partial agonist of PPARγ, although several studies have reported that 9-HODE is a PPARγ agonist [34,35]. PPARγ can accommodate two molecules of 9- and 13-HODEs in the ligand-binding pocket as shown in the chain A of 2VSR [19]. We speculate that the biological activity of 9-HODE as a PPARγ ligand depends on whether PPARγ LBD binds to one or two molecules of 9-HODE.

We performed flexible docking just by imposing the positions of carbon and oxygen atoms in the carboxylate groups of HODE isomers. This allowed us to explore the poses in the ligand-binding pocket of PPARγ, and our observations were consistent with those of previous studies [18,36] that most ligands interact in the canonical ligand-binding pocket of PPARγ at the same level (Table 1). In addition, it is known that the stimulatory effects of 9-HODE and 13-HODE on *aP2* expression can be reproduced by rosiglitazone, and the effects of all three ligands can be inhibited by a specific inhibitor of PPARγ [34]. This may legitimize the use of crystal structures of PPARγ LBD in complex with rosiglitazone in this study. In fact, it was demonstrated that HODE isomers, including not only 9- and 13-HODEs but also 10- and 12-HODEs, directly bound to PPARγ LBD in WaterLOGSY NMR. Further studies are needed to compare the extent of direct affinity between HODE isomers and PPARγ LBD. Our results provide novel findings that 10- and 12-(*Z,E*)-HODEs bind to PPARγ LBD.

In conclusion, we found that stereo-, regio-, and enantio-isomers of HODE showed different PPARγ agonist activities. This is the first report showing that 10- and 12-HODEs specifically generated by singlet oxygen have PPARγ agonist activity at the same level as that of 13-HODEs. It suggests that transient increase in plasma levels of 10- and 12-(*Z,E*)-HODEs in prediabetes mice [4] may be a compensatory change to prevent the progression of diabetes; however, further studies are necessary to clarify the function of 10- and 12-(*Z,E*)-HODEs in the development of diabetes. Our findings will contribute to understanding the *in vivo* biological function of lipid peroxidation products.

## Supplementary Material

Supplementary Figure S1-S2

## Abbreviations

DSS: sodium 2,2-dimethyl-2-silapentane-5-sulfonate
Fluc: firefly luciferase
HODE: hydroxyoctadecadienoic acid
HPODE: hydroperoxyoctadecadienoic acid
LBD: ligand-binding domain
MOE: molecular operating environment
PDB: Protein Data Bank
PGZ: pioglitazone
PPARγ: peroxisome proliferator-activated receptor γ
PUFA: polyunsaturated fatty acid
Rluc: *Renilla reniformis* luciferase
TZD: thiazolidinedione
WaterLOGSY: water-ligand observed via gradient spectroscopy
