# Supplementary material for: Comprehensive analysis of PPARγ agonist activities of stereo-, regio-, and enantio-isomers of hydroxyoctadecadienoic acids
Source: Biosci Rep. 2020 Apr 23;40(4):BSR20193767. doi: 10.1042/BSR20193767 (PMC7198041; doi:10.1042/BSR20193767)
Supplement: Supplementary Figure S1-S2 [file BSR-2019-3767_supp.pdf]

Supplementary figure 1

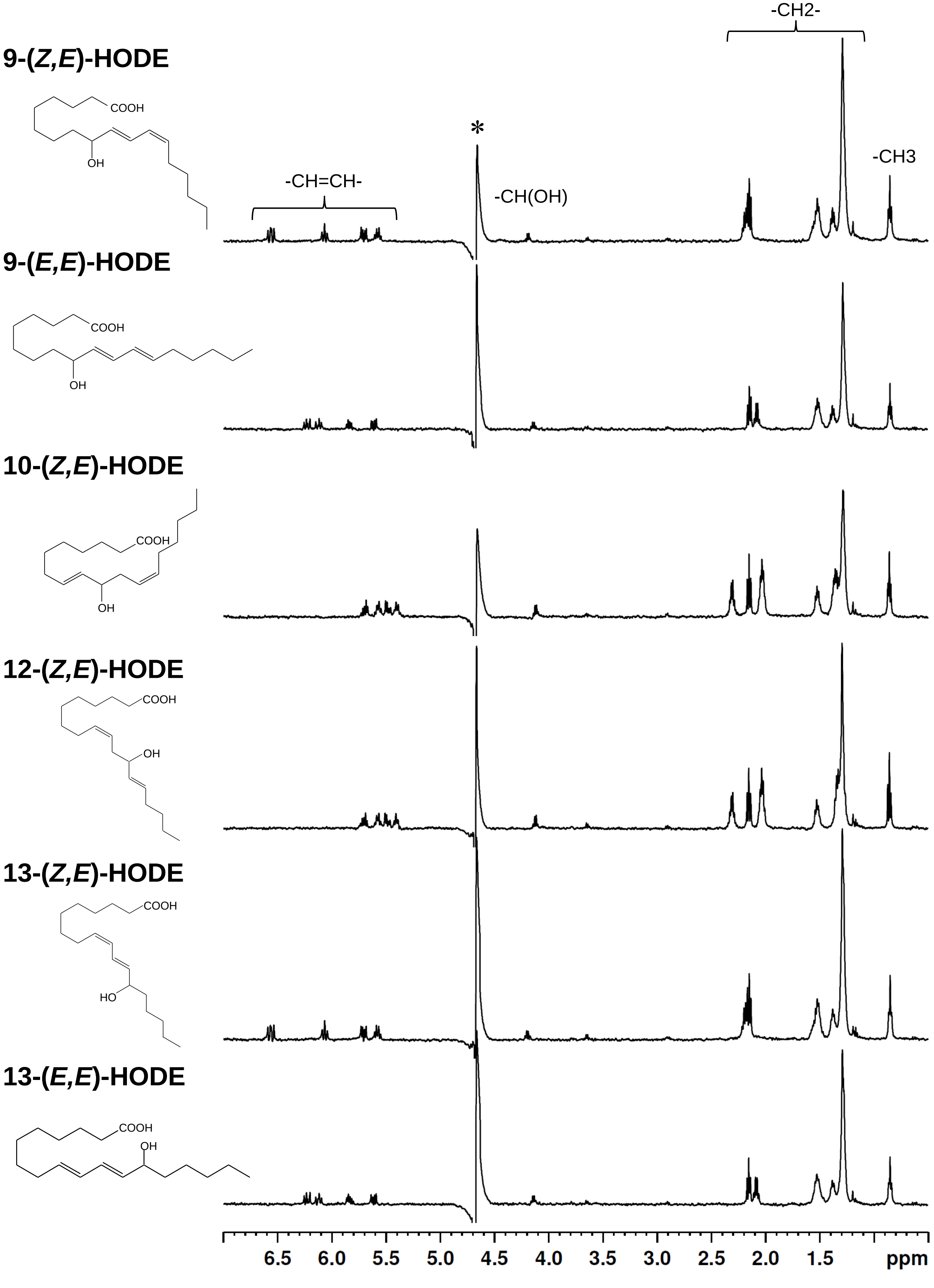

Supplementary Figure 1. ^{1}H-NMR spectra recorded forthe HODEisomers. The temporary assignments areindicated above the signals.An asterisk indicates the position of the water signal.

Supplementary figure 2

WaterLOGSY NMR

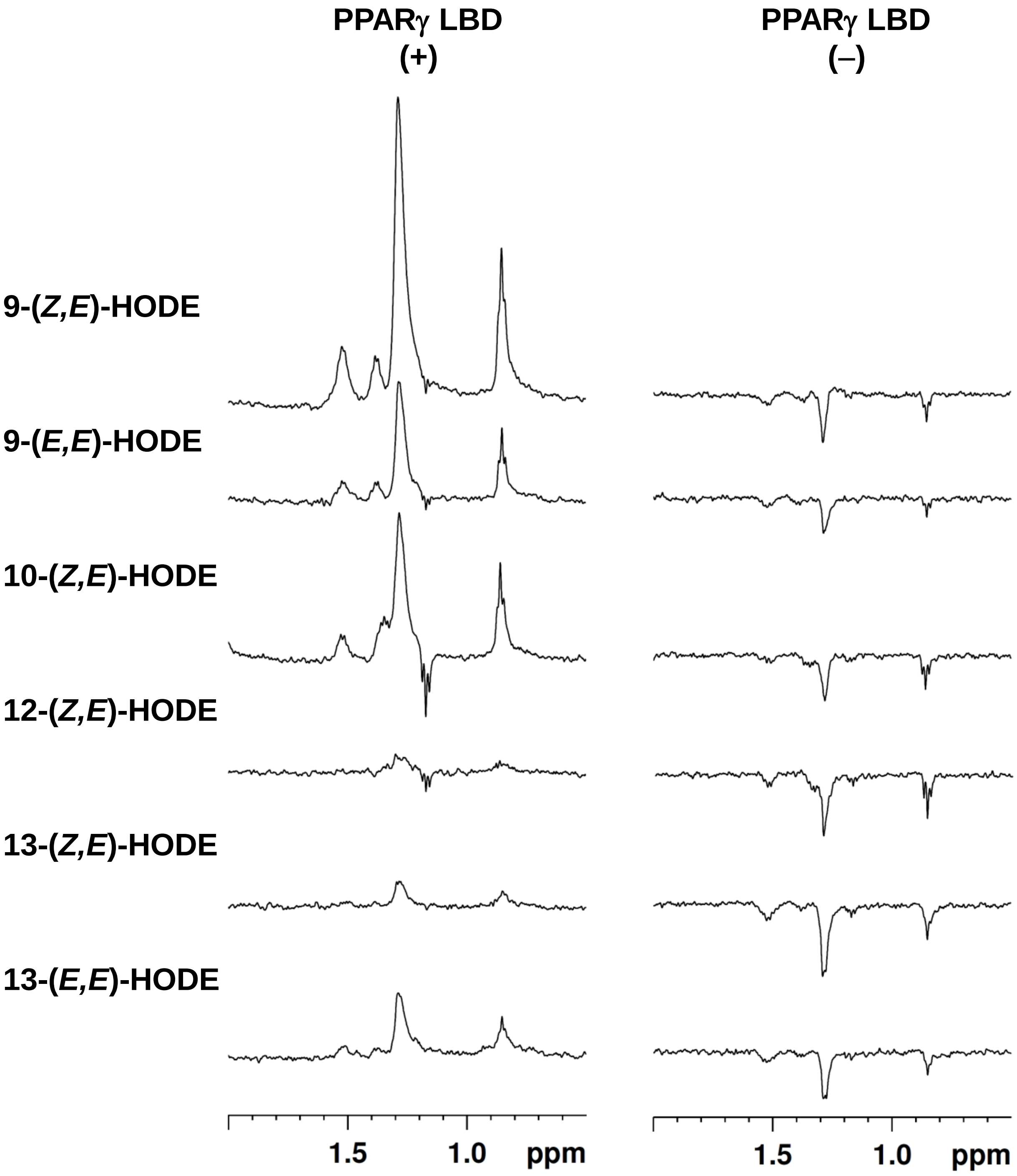

Supplementary Figure 2. WaterLOGSY experiments of the HODE isomers in the presence (left) and absence (right) of PPARγLBD.
